# The Dairy Cow Slurry Composition Used as Organic Fertilizer Is Influenced by the Level and Origin of the Dietary Protein

**DOI:** 10.3390/ani11102812

**Published:** 2021-09-27

**Authors:** Fernando Vicente, Douâa Elouadaf, Alejandra Sánchez-Vera, Ana Soldado, Senén De La Torre-Santos, Adela Martínez-Fernández

**Affiliations:** 1 Servicio Regional de Investigación y Desarrollo Agroalimentario (SERIDA), Carretera AS-267, PK. 19, 33300 Villaviciosa, Spain; douaa.elouadaf@gmail.com (D.E.); sanchezveraalejandra@gmail.com (A.S.-V.); soldadoana@uniovi.es (A.S.); senen_30@msn.com (S.D.L.T.-S.); admartinez@serida.org (A.M.-F.); 2 Facultad de Química, Universidad de Oviedo, Avenida Julián Clavería, 8, 33006 Oviedo, Spain

**Keywords:** protein level, legume silages, nitrogen excretion, ammonia-N, dairy cow

## Abstract

**Simple Summary:**

Dairy cattle is a source of ammonia because only 25–35% of the dietary nitrogen is used for the synthesis of milk, and the remainder is excreted through feces and urine. A reduction in dietary nitrogen is an effective way to decrease nitrogen excretions and subsequent ammonia emissions. However, this reduction should not induce a decrease in the potential yield of the cows. On the other hand, legumes are more susceptible than grasses to undergo proteolysis in the silage process due to their higher protein content. However, not all legumes have the same rate of proteolysis rate. With the main objective of improving the quality of the slurry to be used as organic fertilizer, two sequential experiments were carried out. In the first, it was intended to determine the optimal level of dietary nitrogen intake necessary for high-production dairy cows. Once this level was established, two legume silages with different proteolysis rates were evaluated. In conclusion, dairy cows producing more than 30 kg of milk per day can meet their needs with diets with 13% of protein, reducing nitrogen losses through urine. The main pathway for the excretion of dietary nitrogen provided by legume silage is the urine, and the protein of field pea silage is metabolized towards ammonia production to a larger extent than the protein of faba bean silage.

**Abstract:**

Less than 30% of dairy cattle’s nitrogen ingested is retained in milk. Therefore, large amounts of nitrogen can be excreted in manure and urine with a potential environmental impact. In addition, some legume forages can be more susceptible to proteolysis during the silage process than grasses, and dairy cows fed these legume silages would excrete a larger quantity of nitrogen in slurry. The objectives of this work were to evaluate the amount of nitrogen excretion in dairy cows fed different protein levels and legume silages with a view to improve the slurry quality as a co-product that can be used as fertilizer. Two double 3 × 3 Latin square trials were carried out in order to study three different protein levels (high, medium, and low) and three different silages (grass, faba bean, and field pea). Dry matter intake, milk production, and composition were not affected by treatments. The excretion of ammonia-N in the urine was almost four times lower in the diet with the lowest protein level. The ammonia-N in the urine was twice as high with the pea silage than faba bean and grass silages. In conclusion, the diet containing 13% of protein meets the protein requirement for lactating cows producing 31 kg daily, with low nitrogen excretion in the urine, and the main pathway for the excretion of surplus nitrogen from legume silages is through urine and the metabolization of pea silage protein goes toward ammonia-N.

## 1. Introduction

The evolution of agriculture in the last few decades has been marked by high fertilization, a determining factor in the agricultural productivity increase. However, the intensive production of crops with high-yielding varieties has become a major issue of concern, particularly because nitrogen (N) overfertilization may result in major environmental problems in surface and groundwater quality [1]. Between 50% and 70% of N supplied can be transferred to the ecosystem, causing eutrophication [2] and even biologically dead zones [3]. Environment-friendly alternatives must be considered, and lowering inputs of N fertilizer will be part of the solution, but they should neither lead to a loss of forage production and nutritive value nor compromise the livestock production. In addition, there is a significant deficit of high-quality protein forages grown for livestock in Europe, which makes Europe highly dependent on a foreign protein supply. Currently, Europe imports the equivalent of around 40 million tons of soya beans per year from overseas [4]. Therefore, it would be highly interesting to increase locally adapted protein-rich crops to reduce imported protein feeds for dairy cow rations [5]. This would reduce dependence on feed imports from overseas and would be a chance for rural development.

Legumes could be a good option because they provide a good source of protein for animals with positive effects on the environment, associated with their role in N-fixation, and therefore, a reduction in inorganic N-fertilization [6]. However, only 3–4% of the arable land area in Europe is used for legume crops. A wide range of legume species adapted to different European farming circumstances is available to expand legume production. The Italian ryegrass (*Lolium multiflorum* Lam.) is a common winter crop in rotation with maize (*Zea mays* L.) as the summer crop in many European dairy farms [7]. This crop rotation is very productive but highly demanding on nitrogen and could have negative effects on soil fertility [8] and soil health [9]. Including legumes in this crop system strengthens local economies and increases local protein self-sufficiency. In addition, legumes are a great opportunity to solve those agronomic and environmental challenges due to their high yield, high protein content, improving the edaphic profile, and low nitrogen requirements [10]. The nutrition of high-yield animals with diets of similar composition throughout the year require conserved forage food, including ensiled forages. However, some forage legumes have lower ensilability than grasses due to their higher protein content, lower carbohydrate content, and greater buffering capacity [11], and they are more susceptible to proteolysis during the ensiling process due to massive protein degradation and amino acid deamination [12]. Further, not all legumes have the same proteolysis rate during the ensilage process [13]. Faba bean (*Vicia faba* L.) and pea (*Pisum sativum* L.) are two legumes locally grown in Western Europe that appear to have potential as high-yield forages [14]. Faba bean is considered a forage with a medium–high ensilability [15], while field pea degrades much of its protein to ammonia, amino acids, and peptides during the ensilage process [16].

Ruminants transform ingested vegetable N into animal products, eliminating the unused part in the form of excrements rich in N. However, they have a low-efficiency N utilization. Dairy cattle use up to 25–35% of the dietary N for milk synthesis, while the remainder is excreted through feces and urine [17]. Hence, a large amount of dietary N is excreted into the environment from dairy cattle, mainly (over 60%) as urinary nitrogen [18]. Urea in the urine of cattle accounts for the most part of the urinary N and is easily hydrolyzed to NH_4_^+^, whereas the feces of cattle contain a lower level of rapidly decomposable N than the urine [19]. As a consequence, the development of livestock has led to a concentration of excreted N in the environment when the slurry has been mismanaged. Therefore, decreasing the urinary excretions of N and shifting the N excretion from urine to feces are important ways to mitigate the NH_3_ emissions from dairy cattle. The dietary crude protein level had important impacts on the proportions of N in the feces and urine of dairy cows [20]. Diets with different protein concentrations have similar fecal N excretion but differ in urinary N excretion [21] because fecal N excretion is directly related to animal size and weight [22], while urinary N is the result of the catabolism of body proteins and the surplus of dietary N neither used by the ruminal microbiota nor by the animal after its intestinal absorption [23]. However, at low dietary crude protein levels, the feces are the main pathway for N excretion [24], while at high levels, the urinary N excretion will be increased [25]. A reduction in the dietary protein content appears to be the most effective strategy to decrease N excretion and related NH_3_ emissions [26]. As legumes help reduce cattle urinary N excretion [27], and also have lower requirements of N fertilizer application for their growing, this potentially makes them candidates for strategies to reduce the amount of N flowing through dairy cows, and growing them responds to the political and public pressures on dairying and dairy farmers.

The research hypothesis was that it is possible to reduce the dietary protein of dairy cows to a level compatible with a profitable performance in order to reduce nitrogen excretion through feces and urine. Furthermore, the origin of dietary protein could have an effect on the excretion route of nitrogen surplus. The objective was to evaluate the effect of different protein levels in the ration of dairy cows on the quality of slurries used as fertilizer. Furthermore, having established the optimal level of protein, a second objective was to evaluate the amount of nitrogen excreted by dairy cows fed two legume silages as alternatives to Italian ryegrass silage. Some preliminary results were previously reported [28,29].

## 2. Materials and Methods

Two trials were carried out at the SERIDA experimental farm, located at Villaviciosa, Asturias, Spain (43°28′50″ N, 5°26′27″ W, 10 m above sea level). The research was conducted in accordance with the European Union Animal Welfare Directive Number 2010/63/EU with approval of the Research Ethics Committee of the University of Oviedo (Ref. PRONAE 26-2018).

### 2.1. Animals and Diets

#### 2.1.1. First Trial

Six Holstein dairy cows with 677 ± 37.3 kg of live weight (average ± s.e.m.), and 35.4 ± 6.38 kg milk per day with 60 ± 11 days in milk were housed in a tie-stall barn. Three experimental diets were made daily as total mixed rations. The diets had a 55:45, forage:concentrate ratio, were isocaloric (net energy for lactation basis), and with three crude protein levels: High, Medium, and Low. The daily feed ration was offered in two equal fractions after each milking. Refusals were removed and weighed daily before a fresh ration was offered. A 5% refusal of dry matter per day for each cow was targeted to provide ad libitum access to feed. Additionally, two concentrates were distributed during milking sessions. The ingredients and chemical composition of the total mixed rations are detailed in Table 1. Water was continuously available.

#### 2.1.2. Second Trial

Six Holstein dairy cows with 580 ± 16.6 kg of live weight and 29.9 ± 2.58 kg milk per day with 157 ± 12 days in milk were housed in a tie-stall barn. The diets were based on grass silage, faba bean silage, or field pea silage, made as total mixed rations and formulated to be isocaloric and isonitrogenous with a forage:concentrate ratio of 60:40 (Table 1). The feeding management was the same as the first trial.

### 2.2. Experimental Procedure

#### 2.2.1. First Trial

The cows were randomly assigned to 1 to 3 experimental treatments in a 3 × 3 replicated Latin square design. The treatments consisted of three isoenergetic diets with three crude protein levels: (1) High protein (HP); (2) Medium protein (MP); and (3) Low protein (LP). The evaluation time was 42 days, divided into three periods with 10 initial days of adaptation to diet and four sampling days. Individual dry matter intake and milk production were recorded daily. Diet and refusal were sampled daily in each experimental period during the four sampling days, grouped for each animal and period and frozen until analysis. Feces were also collected daily from the collector box, and a proportional aliquot was taken from each animal (2% of the total daily production) and stored at −20 °C to get a composite sample for each animal and period. Urine was collected daily in 1 L of a sulfuric acid solution (10% *v*/*v*) to minimize ammonia (NH_3_) volatilization by means of external separators of polyethylene vinyl acetate stuck on the vulva with the use of a biocompatible glue. Daily samples were pooled on individual animal bases after recording the weight and the specific gravity, and one sample (1% of the total daily production) was taken and stored at −20 °C to get a composite sample for each animal and period. Cows were milked twice daily at 07:00 and 19:00 h. Milk was sampled from each animal on the second and fourth sampling days. Morning and evening samples from each cow were mixed according to its milk yield on both days to get a representative sample by day and cow.

#### 2.2.2. Second Trial

Cows were randomly assigned to 1 to 3 experimental treatments in a 3 × 3 replicated Latin square design. Treatments consisted of isoenergic and isonitrogenous diets based on: (1) grass silage (GS: control); (2) faba bean silage (FB); and (3) field pea silage (PS). The experimental procedure was the same as the first trial.

### 2.3. Analytical Procedures

The analytical procedures were the same in both trials. Mixed diet and refusal samples were dried (60 °C, 24 h) and milled (0.75 mm). The concentrate samples were ground to 1 mm. Both samples were analyzed by near-infrared spectroscopy (Foss NIRSystem 5000, FOSS NIRSystems, Inc., Laurel, MD, USA) for dry matter (DM), organic matter (OM), crude protein (CP), ether extract (EE), starch, neutral detergent fiber (NDF), and acid detergent fiber (ADF). The net energy for lactation content was estimated in all samples according to NRC [30]. Ammonia N contents of fresh feces and urine were determined by the Kjeldahl method after the alkalinization of samples with magnesium oxide (10% *v*/*v*). The DM of feces was determined by drying samples to a constant weight at 60 °C, and OM by ashing at 550 °C. Total N content was determined by the Kjeldahl method. The ether extract in feces was determined, after previous hydrolysis of the sample with HCl 3M, by extraction in a 2050 Soxtec Avanti equipment (FOSS Tecator Hillerød Denmark) using petroleum ether as the organic solvent. The NDF concentration in feces was determined by the procedures of Van Soest [31]. Milk samples were analyzed for fat, protein, lactose, solids non-fat, and urea contents by Fourier Transform Infrared (MilkoScan FT 6000, FOSS Tecator, Hillerød, Denmark).

### 2.4. Calculations and Statistical Analyses

The calculations and statistical analyses were the same in both trials. The nutrient intakes were calculated by subtracting the nutrient proportion of refusals from the nutrient content of the offered feedstuff. The nitrogen efficiency was calculated from the ratio of N excretion in milk and N ingested with diet. The apparent digestibility coefficients were estimated according to the amount of daily food consumed, daily feces excreted, and the content of each nutrient in both feces and food.

Each dairy cow fed a given treatment at each period was considered the experimental unit in all analyses. The daily mean value for each variable was calculated as the average of four days in each experimental period and was statistically analyzed by an analysis of variance according to a mixed model using software R [32], according to the model: Y_ijk_ = m + T_i_ + P_j_ + (T × P)_ij_ + C_k_ + e_ijk_, where Y_ijk_ was the dependent variable, m the overall mean, and e_ijk_ the residual error. Treatment (T_i_) and period (P_j_) were considered as fixed effects, and cow (C_k_) as the random effect. Differences were considered significant at *p* < 0.05.

## 3. Results

### 3.1. First Trial

The nutrient intakes after the adjustment of intake by nutrient of refusals are shown in Table 2. The daily average dry matter intake was 20.5 ± 1.21 kg/day. Despite the different protein levels offered, there were also no significant differences in the crude protein intake among treatments (3.3, 3.4, and 2.9 kg crude protein/day for HP, MP, and LP treatments, respectively, *p* > 0.05). Both intakes of starch and ether extract were greater (*p* < 0.05) in the high protein diet than medium and low protein treatments.

Milk yield did not show significant differences between treatments (Table 3). However, milk yield decreased around one kilogram for each level that dietary protein decreased (33.0, 31.9, and 30.8 kg milk/day for HP, MP, and LP treatments, respectively, *p* > 0.05). Milk composition also showed no differences among treatments, with average values of 41.7 ± 2.37 g fat/kg milk, 29.4 ± 0.38 g protein/kg milk, 42.6 ± 2.37 g lactose/kg milk, and 222 mg urea/kg milk. This makes that total N excreted by milk decrease according to the diet protein level, though the nitrogen efficiency was similar in all treatments (26.9%, 27.9%, and 31.1% for HP, MP, and LP treatments, respectively, *p* > 0.05).

Table 4 shows the chemical composition of feces and nutrients excreted in feces. The level of dietary protein did not affect the daily excreted amounts of DM, OM, and NDF by feces. Further, the excretions of total N and ammonia N in feces did not differ significantly (*p* > 0.05) among diets, with 230, 219, and 203 g N/day and 18, 14, and 16 g ammonia-N/day for the high, medium, and low protein diets, respectively. The apparent digestibility of DM, OM, and NDF did not differ significantly between the diets (55.80 ± 2.247%, 58.78 ± 1.605%, and 43.95 ± 2.544% for DM, OM, and NDF, respectively). However, the crude protein digestibility was significantly lower (*p* < 0.05) in the low protein treatment (55.71%) than the medium protein treatment (59.28%), while the high protein level showed intermediate values (56.31%, *p* > 0.05).

Table 5 shows the chemical composition of urine and nutrients excreted in urine. The urine had an average of 4% dry matter. The ammonia content of urine was affected (*p* < 0.05) by the level of dietary protein. LP treatment had a lower concentration of ammonia nitrogen than HP treatment, while MP treatment had intermediate values. Further, the level of protein affected (*p* < 0.05) to total and ammonia nitrogen excretions in urine.

### 3.2. Second Trial

According to the results of the first trial, it seems that a protein level in the diet of 13% can meet the dairy cows’ requirements. Therefore, the second trial was made with this level (Table 1). The nutrient intakes after the adjustment of intake by nutrient of refusals are shown in Table 6. There were no differences in any of the nutrients among treatments except in starch and ether extract intakes. Since experimental diets were isonitrogenous (13.6 ± 6.4% CP), the daily nitrogen intake was the same among experimental diets. The intake of ether extract was lower (*p* < 0.05) with diets based on faba bean silage than grass silage or field pea silage. The intake of starch was higher in PS treatment than in GS treatment (*p* < 0.01), while FB treatment showed intermediate values.

Milk yield did not show significant differences among treatments (Table 7). Milk composition was also similar among treatments, except in the urea content. The milk from cows feeding diets based on legume silages had greater concentration of urea than dairy cows feeding the diet based on grass silage (*p* < 0.05). The nitrogen efficiency was similar in all treatments, with an average of 27.7 ± 0.86%.

Table 8 shows the chemical composition of feces and nutrients excreted in feces. The type of silage did affect nutrients excretion. However, the daily ammonia excretion of dairy cows feeding grass silage was slightly higher (*p* > 0.05) than dairy cows feeding on both legume silages. The apparent digestibility of DM and NDF were higher in a diet based on faba beans than diets with grass or field pea silages (62.35% vs. 57.92% and 58.07%, respectively, for DM digestibility, *p* < 0.05, and 58.08% vs. 49.48% and 51.75%, respectively, for NDF digestibility, *p* < 0.05). However, the organic matter digestibility (62.82 ± 0.944%) and crude protein digestibility (57.66 ± 1.036%) were no different among treatments.

Table 9 shows the chemical composition of urine and nutrient excreted in the urine. The urine excreted by dairy cows fed a diet based on field pea silage had a higher ammonia (*p* < 0.01) and nitrogen (*p* < 0.001) concentration in urine than the other treatments. However, their daily urine volume excreted was lower (*p* < 0.05). Furthermore, the total and ammonia nitrogen excreted daily were similar among treatments.

## 4. Discussion

The voluntary intake of DM was similar in all treatments. According to the literature, the dry matter intake increases as the level of crude protein in the diet increases [33,34]. In a similar study comparing diets with 15.1%, 16.7%, and 18.4% of CP, the DM intake was lower at the first level [35]. Further, dairy cows fed a diet of 12% CP ingested 0.8 kg DM/day less than those fed 18% CP [21]. However, in our study, with similar levels of protein, the difference between LP and HP treatments was of 1.07 kg DM/day, and there were no differences in DM intake between MP and HP. Furthermore, in our study, despite the theoretically different protein levels, there were also no statistical differences in the crude protein intake among treatments; the MP treatment even had a higher protein intake than the HP treatment. It might be thought that this is probably due to the capacity of self-regulation of animals. However, there is no evidence that ruminants regulate their requirements or look for specific nutrients, except sodium [36]. No explanation for the abnormal result was found. In the second trial, since experimental diets were isonitrogenous, the daily nitrogen intake was the same among experimental diets. The starch and ether extract intakes were statistically higher in HP treatment than in the others because this diet had a higher proportion of starch and ether extract. Both nutrient intakes, ether extract and starch, also had differences in the second trial. However, ether extract intake differences were negligible (60–70 g/day). The highest starch intake was seen in diets based on legume silages, probably due to the fact that faba bean and the field pea were harvested when both species were in the phenological stage of pods with grain. The apparent digestibility of DM, OM, and NDF was not affected by the protein level as in other studies comparing 15.8% or 18.3% of CP in the diets [37,38]. However, Edouard et al. [21] reported that the animals fed diets with 12% CP had a significant reduction of 2.5% in DM digestibility compared to when fed diets with 18% of CP. However, the apparent digestibility of DM and NDF were affected by the type of silage, with higher values in the diet based on faba bean. Some studies have shown a high digestibility of faba bean [39], probably due to a high ruminal solubility [40]. The apparent digestibility of the protein was significantly lower in the LP treatment, probably due to a low microbial fermentation of the protein into the rumen since the fecal nitrogen remained constant between treatments [41]. The results confirm that a similar fecal nitrogen excretion is obtained with varying levels of crude protein. The nitrogen excreted in feces is derived from non-digestible microbial protein, endogenous protein, and non-digestible dietary proteins. The fecal nitrogen varies with the ingestion of DM, which is correlated with the body size and live weight of the animal and its production level [22].

The protein concentration in milk was smaller than expected for all protein levels. In general, the first limitation to milk protein synthesis is the amount of metabolizable energy available [42]. However, the diets in the first trial were formulated with content of energy according to the requirements for dairy cattle [30]. Thus, the low protein concentration might be due to a decrease in the rate of microbial protein synthesis [43], resulting in decreased rates of synthesis of milk protein [44]. That could be due to a lack of synchronization between energy and ruminal protein degradability, because of the different ingredients of concentrates offered during milking or the different ratio of alfalfa hay and barley straw among diets. However, the synchronized dietary energy and protein to improve production and efficiency have resulted in limited success [45]. The source of protein did not influence the protein content in milk. However, urea content in milk was greater with legume silages than GS. The protein in legume silage is subject to extensive degradation to non-protein nitrogen (NPN) during the silage process. The NPN will be rapidly degraded to ammonia in the rumen and, if not captured as microbial protein, will end up largely as urea-N. The nitrogen efficiency is more than three percentage points higher in the low protein treatment than the medium and high protein treatments because the ingestion of N was also lower in the low protein treatment. Several studies have reported that the nitrogen efficiency rises when on a low-protein diet and falls when on a high-protein diet and maintaining the milk yield [21,46]. In the second trial, the nitrogen efficiency was lower than that seen in the LP treatment of the first trial and similar to HP and MP treatments. The slight decrease in nitrogen efficiency in the second trial could be due to the difference in amino acid profile between the diets of the first and second trials. Proteins from alfalfa hay contain more methionine than grass, faba bean, and field pea silages [30], and methionine is one of the limiting amino acids for milk production. Improved milk nitrogen efficiency has been reported when the dietary supply of methionine is increased [47]. In any case, the nitrogen efficiency value in both trials is within the range described in previous studies [17]. Virtually all of the remaining N will be excreted in the urine (30%) and feces (44%).

When the level of the protein in the diet increased, the N excreted in the urine also increased, both as total nitrogen and ammonia nitrogen [21,38]. However, in our study, the differences between HP and MP treatments are low because the crude protein intake in both treatments was similar, while the LP treatment had the lowest crude protein intake and, consequently, it had the lowest urine N excretion. The shortfall in meeting the three different levels of protein constitutes the main limitation of our study. Nitrogen excreted in the urine results from the catabolism of protein in the body and its surplus in the diet. Its excretion is caused by an excess of degradable nitrogen in the rumen that is not valued by microbes or an excess of amino acids at the intestinal level not valued by the animal [23]. The pea silage treatment had a greater concentration of ammonia nitrogen in urine than other treatments in the second trial. This could be indicative of a high proteolysis rate of pea silage, and the NPN would be rapidly degraded to ammonia in the rumen and finally excreted through the urine. Furthermore, the greater non-ammonia-N urine excretion with legume silages might increase ammonia emissions because all of the nitrogen in the urine would be converted into ammonia nitrogen within few days.

## 5. Conclusions

It is, therefore, concluded that the diet containing 13% of CP meets the protein requirement for lactating cows producing 31 kg of milk per day. The additional nitrogen in higher crude protein treatments resulted in a substantial increase of ammonia nitrogen in the urine and, consequently, in the slurry. A low protein diet could promote the reuse of urea for microbial synthesis, compensating for the deficiency of degradable protein for milk protein production, while diets with a higher protein level could entail higher uremia, which resulted from the production of ammonia in the rumen by the microbes. This extra nitrogen was lost as urea in the urine and could increase the ammonia emissions by the slurry. The pathway for the excretion of dietary nitrogen from legume silages is mainly through urine, and the metabolization of field pea silage protein goes toward ammonia-nitrogen. Therefore, the use of slurry as fertilizer from dairy cows feeding legume silages could increase the emissions of nitrogen to the environment.

## Figures and Tables

**Table 1:** Composition of ingredients (% dry matter basis), nutritive value (g/kg DM), and net energy for lactation (Mcal/kg DM) of the total mixed rations. First trial: high protein (HP), medium protein (MP), low protein (LP). Second trial: grass silage (GS), faba bean silage (FB), field pea silage (PS).

	First Trial			Second Trial		
	HP	MP	LP	GS	FB	PS
Ingredient:						
Italian ryegrass silage	31.9	30.1	30.3	40.1	-	-
Faba bean silage	-	-	-	-	48.1	-
Field pea silage	-	-	-	-	-	47.0
Alfalfa hay	13.3	6.3	6.4	-	-	-
Barley Straw	7.0	17.5	17.6	19.3	14.4	13.1
Compound feedstuff ^1^	34.9	33.8	34.1	36.8	33.5	35.8
Concentrate 1 ^2^	13.0	8.2	-	3.9	4.0	4.2
Concentrate 2 ^3^	-	4.1	11.6	-	-	-
Component:						
Dry matter	460	440	438	371	449	408
Organic matter	891	888	888	895	901	887
Crude protein	176	158	138	134	135	140
Ether extract	29	25	24	29	26	30
Starch	187	144	156	108	110	128
Neutral detergent fiber	424	324	320	491	519	494
Acid detergent fiber	253	283	282	319	299	294
Net energy for lactation	1.59	1.56	1.55	1.38	1.41	1.41

^1^ Concentrate included in the total mixed ration. Ingredients in descending order of the percentages by weight present in the compound feedstuff: Cornflakes, Corn, Soybean meal, Barley, Sunflower meal, Rye, By-pass fat, Soybean hulls, Calcium carbonate, Sodium bicarbonate, Cottonseed, Sugarbeet pulp, Sodium chloride, Dicalcium phosphate. ^2^ Concentrate provided at the time of milking. Ingredients in descending order, of the percentages by weight present in the concentrate 1: Corn, Soybean meal, Wheat, Barley, By-pass fat, Molasses, Sodium bicarbonate, Magnesium oxide, Sodium chloride, Calcium carbonate, Dicalcium phosphate, ^3^ Concentrate provided at the time of milking. Ingredients in descending order, of the percentages by weight present in the concentrate 2: Soybean hulls, Corn. Sugarbeet pulp, Wheat bran, Wheat, Sunflower meal, DDGS, Soybean meal, Barley, Calcium carbonate, Sodium bicarbonate, Sodium chloride, Magnesium oxide.

**Table 2:** Nutrient intake (kg/day, dry matter basis) by dairy cows feeding diets with high protein (HP), medium protein (MP), and low protein (LP) levels.

Intake	HP	MP	LP	SEM	*p*-Value
Dry matter	20.78	20.98	19.71	1.206	NS
Organic matter	18.51	18.50	17.46	1.090	NS
Crude protein	3.28	3.35	2.86	0.181	NS
Ether extract	0.70 ^a^	0.62 ^b^	0.65 ^b^	0.034	*
Starch	3.29 ^a^	2.64 ^b^	2.32 ^b^	0.196	*
Neutral detergent fiber	8.81	9.54	8.97	0.581	NS
Acid detergent fiber	5.26	5.89	5.51	0.386	NS

SEM, standard error of means. NS, not significant. ^a, b^: Means in a row with different superscripts differ among treatments. NS: *p* > 0.05, *: *p* < 0.05.

**Table 3:** Production (kg/day) and composition (g/kg) of milk from dairy cows feeding diets with high protein (HP), medium protein (MP), and low protein (LP) levels.

Component	HP	MP	LP	SEM	*p*-Value
Milk yield	33.0	31.9	30.8	2.57	NS
Fat	41.4	41.1	42.7	2.37	NS
Protein	29.8	29.5	29.0	0.38	NS
Lactose	44.6	42.4	40.7	3.54	NS
Solids non-fat	77.9	71.3	71.3	7.06	NS
Urea (mg/kg)	236	270	160	49.8	NS

SEM, standard error of means. NS, not significant.

**Table 4:** Composition of feces (% dry matter basis) and feces excretion (kg/day, dry matter basis) by dairy cows feeding diets with high protein (HP), medium protein (MP), and low protein (LP) levels.

	HP	MP	LP	SEM	*p*-Value
Composition:					
Dry matter	13.16	12.80	12.90	0.935	NS
Organic matter	81.38	82.11	81.61	1.600	NS
Nitrogen	2.54	2.38	2.29	0.155	NS
Ammonia N (g/100 g N)	8.20	6.38	7.77	4.840	NS
Ether extract	2.01	1.74	1.43	0.230	NS
Neutral detergent fiber	54.72	57.26	56.93	1.833	NS
Excretion:					
Dry matter	8.97	9.25	8.88	0.586	NS
Organic matter	7.43	7.67	7.38	0.511	NS
Nitrogen (g/d)	229.58	219.36	203.19	14.231	NS
Ammonia N (g/d)	18.22	13.77	15.95	4.452	NS
Ether extract	0.18 ^a^	0.16 ^ab^	0.13 ^b^	0.019	*
Neutral detergent fiber	5.30	5.06	4.92	0.434	NS

SEM, standard error of means. NS, not significant. ^a, b^: Means in a row with different superscripts differ among treatments. NS: *p* > 0.05, *: *p* < 0.05.

**Table 5:** Composition of urine and urine excretion by dairy cows on feeding diets with high protein (HP), medium protein (MP), and low protein (LP) levels.

	HP	MP	LP	SEM	*p*-Value
Composition:					
Dry matter (%)	4.05	3.61	3.94	0.229	NS
Total Nitrogen (g/100 mL)	0.57	0.61	0.47	0.072	NS
Ammonia N (g/100 mL)	10.55 ^a^	7.62 ^ab^	2.76 ^b^	3.935	*
Excretion:					
Liters/day	25.21	24.35	23.76	1.256	NS
Total Nitrogen (g/d)	143.70 ^a^	148.53 ^a^	111.67 ^b^	9.565	*
Ammonia N (g/d)	2.66 ^a^	1.85 ^ab^	0.66 ^b^	0.601	*

SEM, standard error of means. NS, not significant. ^a, b^: Means in a row with different superscripts differ among treatments. NS: *p* > 0.05, *: *p* < 0.05.

**Table 6:** Nutrient intake (kg/day, dry matter basis) by dairy cows’ feeding diets based on grass silage (GS), faba bean silage (FB), or field pea silage (PS).

Intake	GS	FB	PS	SEM	*p*-Value
Dry matter	16.58	17.56	16.98	0.414	NS
Organic matter	14.85	15.84	15.09	0.369	NS
Crude protein	2.29	2.44	2.45	0.050	NS
Ether extract	0.51 ^a^	0.46 ^b^	0.52 ^a^	0.034	*
Starch	2.05 ^b^	2.13 ^ab^	2.44 ^a^	0.069	**
Neutral detergent fiber	7.87	8.84	8.08	0.246	NS
Acid detergent fiber	5.09	5.06	4.78	0.148	NS

SEM, standard error of means. NS, not significant. ^a, b^: Means in a row with different superscripts differ among treatments. NS: *p* > 0.05, *: *p* < 0.05, **: *p* < 0.01.

**Table 7:** Production (kg/day) and composition (g/kg) of milk from dairy cows with feeding diets based on grass silage (GS), faba bean silage (FB), or field pea silage (PS).

Component	GS	FB	PS	SEM	*p*-Value
Milk yield	21.0	21.7	21.6	7.22	NS
Fat	35.4	34.1	33.9	2.53	NS
Protein	31.0	32.3	31.2	0.52	NS
Lactose	48.1	48.7	47.5	0.40	NS
Solids non-fat	86.4	87.8	85.9	0.68	NS
Urea (mg/kg)	237 ^b^	298 ^a^	281 ^a^	11.6	*

SEM, standard error of means. NS, not significant. ^a, b^: Means in a row with different superscripts differ among treatments. NS: *p* > 0.05, *: *p* < 0.05.

**Table 8:** Composition of feces (% dry matter basis) and feces excretion (kg/day, dry matter basis) by dairy cows fed diets based on grass silage (GS), faba bean silage (FB), or field pea silage (PS).

	GS	FB	PS	SEM	*p*-Value
Composition:					
Dry matter	13.68	14.83	14.38	0.226	NS
Organic matter	82.88	83.55	80.03	0.666	NS
Nitrogen	2.32	2.49	2.21	0.036	NS
Ammonia N (g/100 g N)	7.05	4.92	5.28	0.677	NS
Ether extract	2.00	1.95	1.99	0.041	NS
Neutral detergent fiber	56.91	56.07	54.80	0.581	NS
Excretion:					
Dry matter	6.94	6.72	7.12	0.154	NS
Organic matter	5.75	5.62	5.69	0.116	NS
Nitrogen (g/d)	161.27	167.49	157.71	4.344	NS
Ammonia N (g/d)	11.37	8.38	8.30	1.137	NS
Ether extract	0.14	0.13	0.14	0.005	NS
Neutral detergent fiber	3.95	3.77	3.90	0.085	NS

SEM, standard error of means. NS, not significant.

**Table 9:** Composition of urine and urine excretion by dairy cows fed diets based on grass silage (GS), faba bean silage (FB), or field pea silage (PS).

	GS	FB	PS	SEM	*p*-Value
Composition:					
Dry matter (%)	8.63	7.58	8.57	0.307	NS
Total Nitrogen (g/100 mL)	0.63 ^b^	0.75 ^b^	0.89 ^a^	0.034	***
Ammonia N (g/100 mL)	3.08 ^b^	2.67 ^b^	5.90 ^a^	0.487	**
Excretion:					
Liters/day	17.55 ^a^	16.60 ^a^	13.48 ^b^	0.641	*
Total Nitrogen (g/d)	109.31	124.51	119.13	3.931	NS
Ammonia N (g/d)	0.55	0.44	0.43	0.082	NS

SEM, standard error of means. NS, not significant. ^a, b^: Means in a row with different superscripts differ among treatments. NS: *p* > 0.05, *: *p* < 0.05, **: *p* < 0.01, ***: *p* < 0.001.

## Data Availability

All data used to support the findings of this study are included within the article.
